# Structural imaging predictors of ketamine response in treatment-resistant depression: a machine learning approach

**DOI:** 10.1038/s41398-026-04085-4

**Published:** 2026-05-12

**Authors:** Linda Bryant, Laith Alexander, Sergio Mena, Yael Jacob, Jenna Jubeir, Mu Li, Philipp T. Neukam, Laurel S. Morris, James W. Murrough, Rebecca Price, Nikolaos Koutsouleris, Mitul A. Mehta, Mario Juruena, Fiona Coutts, Paris Alexandros Lalousis

**Affiliations:** 1 Department of Psychosis Studies, Institute of Psychiatry, Psychology and Neuroscience, King’s College London, London, UK; 2 Department of Neuroimaging, King’s College London, London, UK; 3 Department of Psychiatry and Psychotherapy, Ludwig-Maximilians-University, Munich, Germany; 4 Department of Psychiatry, Icahn School of Medicine at Mount Sinai, New York, NY USA; 5 Nash Family Department of Neuroscience & Friedman Brain Institute, Icahn School of Medicine at Mount Sinai, New York, NY USA; 6 BioMedical Engineering and Imaging Institute, Department of Radiology, Icahn School of Medicine at Mount Sinai, New York, NY USA; 7 Nuffield Department of Clinical Neurosciences, University of Oxford, Oxford, UK; 8 Depression and Anxiety Center for Discovery and Treatment, Department of Psychiatry, Icahn School of Medicine of Mount Sinai, New York, NY USA; 9 VISN 2 Mental Illness Research, Education, and Clinical Center (MIRECC), James J. Peters VA Medical Center, Bronx, NY USA; 10 Department of Psychiatry, University of Pittsburgh School of Medicine, Pittsburgh, PA USA; 11 Max Planck Institute of Psychiatry, Munich, Germany; 12 German Center for Mental Health (DZPG), partner site, Munich-Augsburg, Germany; 13 Department of Neuroimaging, Institute of Psychiatry, Psychology and Neuroscience, King’s College London, London, UK; 14 Department of Psychological Medicine, Institute of Psychiatry, Psychology and Neuroscience, King’s College London, London, UK

**Keywords:** Depression, Predictive markers

## Abstract

Ketamine has demonstrated rapid antidepressant efficacy in treatment-resistant depression (TRD), but clinical decision-making is challenging due to variability in individual response. Current trial-and-error prescribing practices may expose patients to ineffective treatment and avoidable adverse effects, underscoring the need for reliable predictive tools to optimize treatment selection and support personalized, evidence-based care. We developed a machine-learning model (support vector classifier) to predict antidepressant response to ketamine using pre-treatment structural MRI data. The model was trained on 99 adults with TRD given a single intravenous ketamine infusion (0.5 mg/kg). Clinical response was defined as a ≥50% reduction in MADRS scores 24 h post-infusion. Internal validation used repeated nested cross-validation, and generalizability was tested in two independent ketamine-treated cohorts (*n* = 51) and a saline-treated control group (*n* = 49). Among ketamine-treated participants, 52 (52.5%) responded to treatment. The model achieved a balanced accuracy of 72.2% (sensitivity = 72.3%, specificity = 73.1%, AUC = 0.72) in the discovery sample and 60.0% (*p* = 0.01, AUC = 0.65) in external validation. Greater gray matter volume in frontal regions predicted response, whereas greater cerebellar volume predicted non-response. Performance dropped to chance in the saline cohort (BAC = 41.1%, AUC = 0.45), supporting pharmacologic specificity. These findings present the first machine-learning model for the prediction of ketamine response in TRD using structural neuroimaging and highlight its potential utility for stratified treatment planning and biomarker-informed interventions while providing mechanistic insight into neuroanatomical predictors of antidepressant response.

## Introduction

Treatment-resistant depression (TRD), commonly defined as major depressive disorder (MDD) unresponsive to at least two adequate antidepressant trials, presents a significant clinical challenge [1–4]. Up to 60% of MDD patients fail to respond to initial pharmacotherapy, and many continue to experience persistent symptoms despite multiple subsequent interventions [1, 5, 6]. Current treatment approaches rely heavily on sequential medication trials; a trial-and-error process that delays effective intervention and increases the risk of chronicity and suicide [1, 6–10].

In recent years, ketamine, an N-methyl-D-aspartate (NMDA) receptor antagonist, has emerged as a novel intervention for TRD, producing rapid antidepressant effects through proposed mechanisms including modulation of glutamatergic signaling and enhancement of synaptic plasticity [11–14]. Following a single subanesthetic intravenous infusion, nearly half of patients demonstrate a significant clinical response within 24 h [12, 15, 16]. However, response is heterogeneous: while some patients achieve near-complete remission, others show minimal or transient benefit, and a subset experience adverse effects such as dissociation or symptom worsening [11, 17, 18].

The factors underlying response heterogeneity remain unclear [15, 16]. In the absence of reliable predictors, clinicians cannot determine in advance which patients are likely to benefit from ketamine— potentially exposing some individuals to a treatment that offers no therapeutic gain, may carry adverse effects, and incurs substantial cost [11, 15, 19]. This variability highlights the need for predictive tools capable of identifying likely responders prior to treatment. Accurate stratification could optimize patient selection, reduce unnecessary exposure, and guide the development of more targeted interventions. For example, early identification of likely responders could help avoid unnecessary exposure to ketamine in non-responders while enabling more timely intervention in high-risk individuals, including those with acute suicidality [20, 21]. Importantly, predicting short-term response to ketamine may offer the highest clinical utility. Ketamine’s rapid antidepressant effects typically emerge within 24 h and may inform decisions about continued treatment or alternative strategies [12, 22, 23].

Previous research has explored various biological and clinical predictors of ketamine response, including peripheral inflammatory markers, cognitive processing speed, and dissociative symptoms [24–26]. However, these variables generally yield low individual-level predictive power and have not translated into actionable models [24, 26]. More recently, neuroimaging studies have highlighted potential structural and functional differences between responders and non-responders in regions such as the anterior cingulate cortex, hippocampus, and prefrontal cortex, suggesting a neuroanatomical basis for response heterogeneity [27–29]. Still, findings remain inconsistent—likely due to small samples, methodological heterogeneity, and the limitations of conventional statistical approaches in capturing complex brain-behavior relationships [27, 30].

Structural magnetic resonance imaging (sMRI) offers a non-invasive opportunity for investigating treatment-relevant brain morphology. Unlike functional MRI (fMRI) or electroencephalography (EEG), sMRI yields stable, trait-like morphometrics that can be standardized across sites and integrated into clinical workflows [27, 30]. Systematic reviews have reported baseline volumetric differences in the anterior cingulate cortex and hippocampus as candidate predictors of antidepressant response to ketamine, but effect directions vary across studies, and no reproducible sMRI biomarker has been established to date [31–34].

To address these gaps, machine learning (ML) offers a data-driven framework capable of identifying high-dimensional, multivariate patterns that may better predict clinical outcomes [30, 35]. ML algorithms can identify latent patterns across multiple brain regions that may distinguish responders from non-responders, with reported accuracies exceeding those of conventional regression-based approaches [36, 37]. Predictive modeling of treatment response across neuroimaging modalities has shown promise in depression [36, 38], but in ketamine specifically, existing ML studies remain limited. Previous efforts typically relied on a narrow set of pre-selected predictors, were constrained by small sample sizes, and employed only internal cross-validation without external test sets [39, 40]. To date, no study has applied ML to pre-treatment structural MRI to predict treatment response to ketamine in TRD specifically.

In this study, we aimed to develop a supervised ML model trained on regional gray matter volume (GMV) parcellations derived from pre-treatment sMRI to predict short-term binary response to ketamine in patients with TRD. We chose to predict binary classification of response, as it offers a clinically interpretable endpoint that aligns with ketamine’s time course of action and treatment decision timeline. We hypothesized that the model would distinguish responders from non-responders with above chance accuracy, and demonstrate external generalizability in independent TRD samples. Our findings aim to advance individualized treatment planning and offer mechanistic insight into the neuroanatomical substrates of ketamine response.

## Materials and methods

### Study design and participants

Our discovery sample used data from a single-site, double-blind, placebo-controlled randomized clinical trial of intravenous ketamine in adults with TRD (NCT03237286) [41]. Participants were randomized to receive a single IV infusion of ketamine (0.5 mg/kg) or saline over 40 min. Only patients from the ketamine-treated arm were included in model development. Clinical and demographic information were recorded at baseline. A total of 103 participants underwent pre-treatment MRI scanning; after quality control of imaging data (described below), 4 participants were excluded for poor image quality, yielding a final neuroimaging sample of 99.

Participants were aged 18 to 65 years and met DSM-5 criteria for major depressive disorder with non-response to at least one adequate antidepressant trial. Further inclusion and exclusion criteria are available on ClinicalTrials.gov (NCT03237286) and summarized in the supplement.

The external validation sample used data from two independent U.S.-based trials. From NCT00088699, 22 participants who received a single ketamine infusion and had available baseline sMRI and pre-/post-infusion MADRS data were included [12]. From NCT00768430, an additional 29 patients meeting the same criteria were included [22]. This yielded a pooled external validation cohort of 51 ketamine-treated TRD participants. Protocol details and eligibility criteria for both trials are available on ClinicalTrials.gov; brief trial descriptions and registry links are provided in the supplement.

### MRI acquisition and image preprocessing

Baseline structural T1-weighted MRI scans were acquired prior to infusion using one of three 3 T Siemens PRISMA scanners located at a single facility. Preprocessing was performed using the Computational Anatomy Toolbox (CAT12, version r2170) implemented within Statistical Parametric Mapping (SPM12) in MATLAB (version R2018b) for both the discovery and validation samples. Preprocessing included probabilistic tissue segmentation into gray matter, white matter, and cerebrospinal fluid using CAT12’s adaptive MAP approach with partial-volume estimation; spatial normalization to MNI space with Jacobian modulation of tissue maps to preserve local volume; and smoothing with an 8 mm Gaussian kernel. Image quality was assessed using the CAT12 image quality rating (IQR) metric; scans with an IQR below C were excluded, yielding a final sample with and average IQR of B.

From each processed image, regional gray-matter volume (GMV) features were derived from the modulated, normalized gray-matter maps produced by CAT12 (standard Voxel-based Morphometry preprocessing). Regional GMV values were summarized within 136 regions of interest from the Neuromorphometrics atlas and used as input features for the machine-learning analyses [42]. Although CAT12 can also generate surface-based morphometry measures (projection-based cortical thickness and surface area), these were not included here; we focused on volumetric GMV to obtain whole-brain (cortical and subcortical) coverage with an appropriate feature dimensionality for our models.

We additionally derived each subject’s total intracranial volume (TIV) to use as a covariate, since head size can affect raw brain volumes. We also included a site covariate corresponding to the MRI acquisition protocol version for each scan (a binary indicator distinguishing two slightly different scanner sequences used over the course of the study), as well as participants’ age and sex, as additional covariates. By accounting for these variables in our analysis, we aimed to isolate neuroanatomical differences related to treatment outcome rather than confounds such as age or scanner parameters.

### Outcome definition

Antidepressant response was defined as a ≥ 50% reduction in MADRS scores from pre-infusion to 24 h post-infusion, consistent with prior ketamine studies [12, 15, 22, 23, 41]. MADRS percentage change was calculated as:

$$\Delta {MADRS} \% =\frac{{MADR}{S}_{{post}}-{MADR}{S}_{{pre}}}{{MADR}{S}_{{pre}}}\times 100$$

Patients were categorized into response or nonresponse groups and these served as outcome labels for classification. Of the 99 patients in the discovery sample, 52 met response criteria (responders), while 47 did not (non-responders), and these group labels were used as classification outcomes.

### Machine learning analysis

**Fig. 1:**
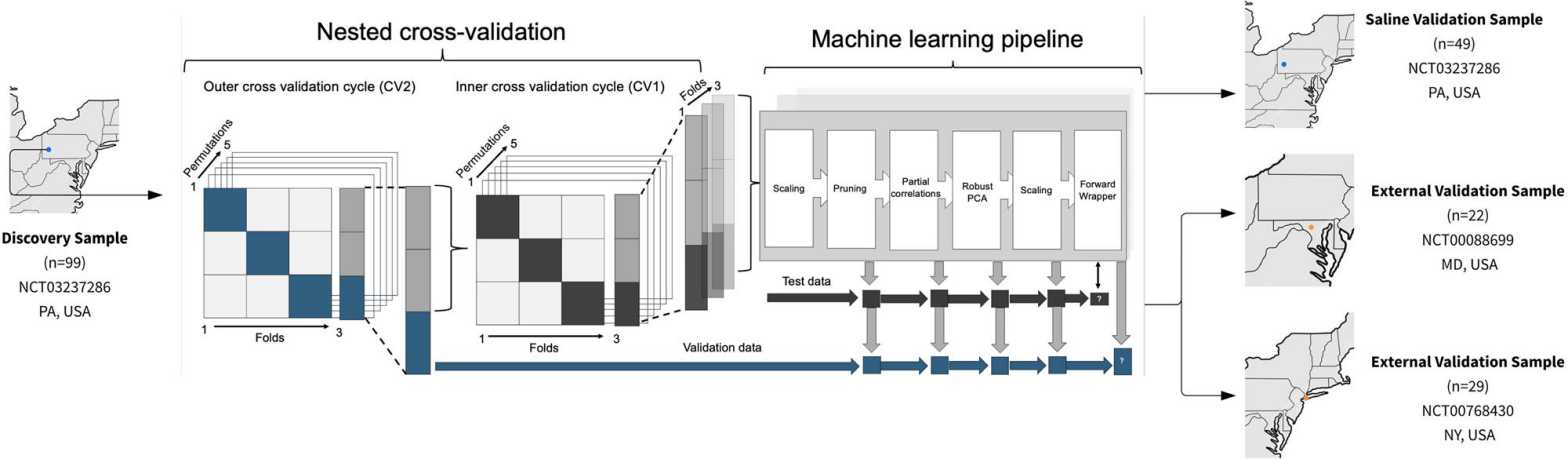
Machine learning analysis design. The discovery model was trained on data from a single-site ketamine trial (NCT03237286) using nested, repeated cross-validation. The outer loop (CV2: 3-fold, 5 repetitions) provided validation data for unbiased performance estimation, while the inner loop (CV1: 3-fold, 5 repetitions) was used for hyperparameter tuning and feature selection. The preprocessing pipeline included scaling, pruning, partial correlation to control for age, sex, site, and TIV, dimensionality reduction via robust PCA, and forward feature selection. Model generalizability was assessed through external validation on two independent single-dose ketamine cohorts (NCT00088699 and NCT00768430; total *n* = 51), and mechanistic specificity was tested on a saline-treated cohort (*n* = 49) from the same trial as the discovery sample.

All machine learning analyses were performed using NeuroMiner (v1.3; GitHub [https://github.com/neurominer-git/NeuroMiner-1]). Model training, preprocessing and hyperparameter optimization were performed within a repeated nested cross-validation (NCV) framework to avoid overfitting and ensure generalizability. The outer loop (CV2) used 3-fold cross-validation repeated 5 times. Each CV2 training set was further subdivided into an inner loop (CV1) using 3-fold cross-validation repeated 5 times for hyperparameter tuning and feature optimization. Figure 1 illustrates the NCV structure and model development pipeline.

The GMV parcellations were preprocessed by first scaling to the range [−1, 1], and removing non-informative features (e.g., zero variance, NaN). Partial correlations were used to regress out covariates (age, sex, scanner sequence, and TIV): and robust principal component analysis (RobPCA) reduced dimensionality to 13 components [43]. A second scaling step was performed post-PCA to ensure all principal components had equal range. Feature selection was conducted using a forward greedy wrapper, iteratively retaining (in steps of 10%) the top 20% of features that contributed to model performance.

Following pre-processing, a linear support vector machine classifier (SVC) was trained to classify responders versus non-responders. The SVC employed a linear solver with L2-regularized L2-loss, dual formulation, no kernel, and a tolerance of 0.01, with hyperplane weighting to account for class imbalances. The regularization parameter (C) was optimized over 11 values (0.0156, 0.0312, 0.0625, 0.1250, 0.2500, 0.5000, 1, 2, 4, 8, and 16). To evaluate the statistical significance of model performance, we conducted label permutation testing with 1000 iterations and assessing performance using BAC, sensitivity, specificity, and AUC [44].

### Model interpretability, specificity testing, and external validation

To assess the neuroanatomical features contributing most strongly to classification, feature weights were analyzed across folds. To interpret regional contributions, model weights were back-projected from the PCA-transformed space to the original GMV feature space, enabling identification of the most predictive brain regions. Consistency and robustness of each predictor were quantified using cross-validation ratios (CVRs), calculated as the mean weight divided by its standard error across folds, and permutation-derived sign-based consistency tests, controlling for false discovery rate (FDR). The CVR measures the stability, magnitude and effect of each feature [45].

To evaluate whether the model learned patterns specific to ketamine-related response, the trained model was applied to the independent saline-treated cohort (*n* = 49) from the same trial as the discovery cohort. A reduction in predictive performance in this cohort would support specificity to ketamine mechanisms. The model was then applied to the pooled external validation sample to determine model generalizability. Scanning protocols varied across datasets, but no harmonization was applied, to emulate real clinical use of our model; instead, site/sequence effects were addressed earlier via covariate regression within the nested cross-validation framework.

### Post hoc transcriptomic annotation of predictive regions

We leveraged the abagen toolbox [46] to extract transcriptomic data from the Allen Human Brain Atlas (AHBA), which includes normalized microarray expression profiles for approximately 20,000 genes sampled from six adult human donors [47]. Using the Neuromorphometrics atlas aligned to MNI space, we focused on the six brain regions that emerged as the top structural predictors of ketamine response in our machine learning model. Upstream QC steps included probe filtering, donor consolidation, and gene/sample normalization per default abagen settings [48]. We specifically extracted expression levels for seven receptor genes—NMDA receptor subunits (GRIN1, GRIN2A, GRIN2B), AMPA receptor subunits (GRIA1, GRIA2), and GABA_A receptor subunits (GABRA1, GABRB2)—selected based on their established pharmacological relevance to ketamine’s mechanism of action [49, 50].

### Ethics approval and consent to participate

The data used in this study were obtained from previously conducted clinical trials (NCT03237286, NCT00088699, and NCT00768430), each of which received approval from the relevant institutional review boards/ethics committees. Written informed consent was obtained from all participants in the original studies.

## Results

### Sample characteristics

Demographic and clinical characteristics for all cohorts are summarized separately in Table 1. The discovery sample had a mean (SD) age of 34.81 (11.01) years, with 62 (62.6%) female and 37 male (37.4%) participants. 52 patients (52.5%) were classified as responders and 47 (47.5%) as non-responders. Responders and non-responders had no difference in age (34.8 ± 11.7 vs. 34.8 ± 10.3 years) or sex distributions (65.4 vs. 59.6% female, respectively). Baseline MADRS scores were comparable across groups (~32), while post-treatment scores differed substantially (mean = 9.1 for responders vs. 24.9 for non-responders).

**Table 1:** Demographic and Clinical Characteristics Across samples.

Sample	Group	n	Female, n (%)	Male, n (%)	Age, mean (SD)	MADRS Pre, mean (SD)	MADRS Post, mean (SD)	MADRS Change, mean (SD)
Discovery	Responders	52	34 (65.4%)	18 (34.6%)	34.84 (11.72)	32.15 (5.56)	9.48 (5.54)	−22.67 (5.43)
	Non-responders	47	28 (59.6%)	19 (40.4%)	34.77 (10.29)	33.34 (5.13)	25.17 (6.72)	−8.00 (6.06)
Saline	Responders	12	7 (58.3%)	5 (41.7%)	29.37 (6.49)	33.92 (5.38)	12.42 (3.60)	−21.50 (6.30)
	Non-responders	37	24 (64.9%)	13 (35.1%)	35.44 (10.66)	31.97 (5.03)	27.24 (7.14)	−4.73 (5.06)
External validation – MD	Responders	5	2 (60.0%)	3 (40.0%)	39.60 (5.41)	31.40 (4.62)	7.20 (6.34)	−24.20 (7.16)
	Non-responders	17	11 (64.7%)	6 (35.3%)	34.59 (9.64)	32.76 (5.02)	27.47 (6.86)	−5.29 (7.67)
External validation – NY	Responders	16	7 (43.8%)	9 (56.3%)	46.34 (16.39)	30.56 (5.40)	8.50 (5.16)	−22.06 (5.81)
	Non-responders	13	5 (38.5%)	8 (61.5%)	41.44 (15.32)	31.46 (5.82)	25.46 (5.65)	−6.00 (4.16)

Each cohort is stratified by treatment response status (responders vs non-responders). Values are presented as mean (SD) for continuous variables and number (%) for categorical variables. MADRS = Montgomery–Åsberg Depression Rating Scale. Clinical response was defined as ≥ 50% reduction in MADRS scores 24 h post-infusion. Change score calculated as post-ketamine – pre-ketamine. Discovery sample from NCT03237286; Saline sample from the same trial; External validation – MD from NCT00088699; External validation – NY from NCT00768430.

The saline-treated validation sample (mean (SD) age, 33.84 (9.78) years; 31 female (63.3%); 18 male (36.7%)) included 12 responders (24.5%) and 37 (75.5%) non-responders.

The pooled external validation sample (mean (SD) age, 42.84 (14.70) years; 25 female (49.0%), 26 male (51.0%)) included 21 responders (41.2%) and 30 non-responders (58.8%).

### Machine learning analyses

#### Classification performance in discovery sample

The SVC trained on pre-treatment GMV parcellations achieved a BAC of 72.2%, sensitivity of 72.3%, specificity of 73.1%, and an AUC of 0.72. Permutation testing confirmed statistical significance (*p* < 0.001). Performance metrics are detailed in Table 2.

**Table 2:** Classification performance metrics across discovery, saline validation, and external validation cohorts.

	TP, n	TN, n	FP, n	FN, n	CCR, %		BAC, %	PV, %		AUC
					Non- responders	Responders		PPV	NPV	
Discovery model	34	38	14	13	72.3	73.1	72.7	70.8	74.5	0.73
Saline validation	15	5	7	22	40.5	41.7	41.1	68.2	18.5	0.45
External validation	16	14	7	14	53.3	66.7	60.0	69.6	50.0	0.65

Performance is reported using confusion matrix counts—true positives (*TP*), true negatives (*TN*), false positives (*FP*), and false negatives (*FN*)—alongside correct classification rate (*CCR*), balanced accuracy (*BAC*) positive predictive value (*PPV*), negative predictive value (*NPV*), and area under the receiver operating characteristic curve (*AUC*).

#### Feature importance and regional contributions

**Fig. 2:**
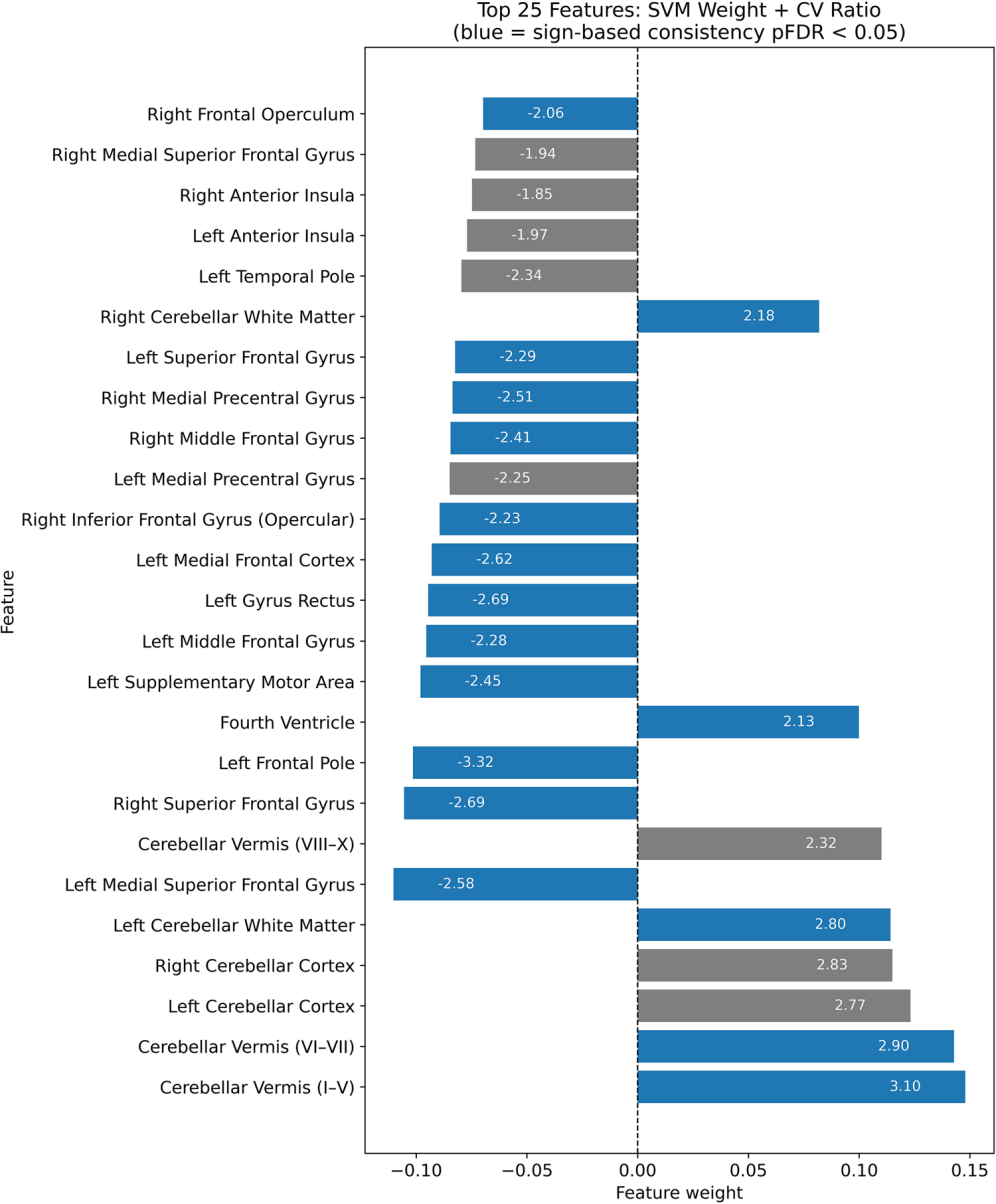
Top 25 features contributing to classification of ketamine response. Bars represent feature weights, with negative weights indicating features that contributed to classification as responders and positive weights indicating features that contributed to classification as non-responders, based on the model’s class coding. Features are ordered by the absolute magnitude of their weight, with the most influential features shown at the bottom. Bar color reflects statistical significance of sign-based consistency across cross-validation folds: blue bars denote features with a consistent direction of contribution across folds at FDR-corrected *p* < 0.05, while grey bars did not meet this threshold. CVRs, annotated inside each bar (values rescaled ×5 for visualization only), quantify the robustness of each feature’s contribution across the inner folds of the nested cross-validation framework. The CV ratio was calculated as the mean weight divided by the standard error of that weight across folds, serving as an interpretable measure of feature stability akin to a standardized effect size.

**Fig. 3:**
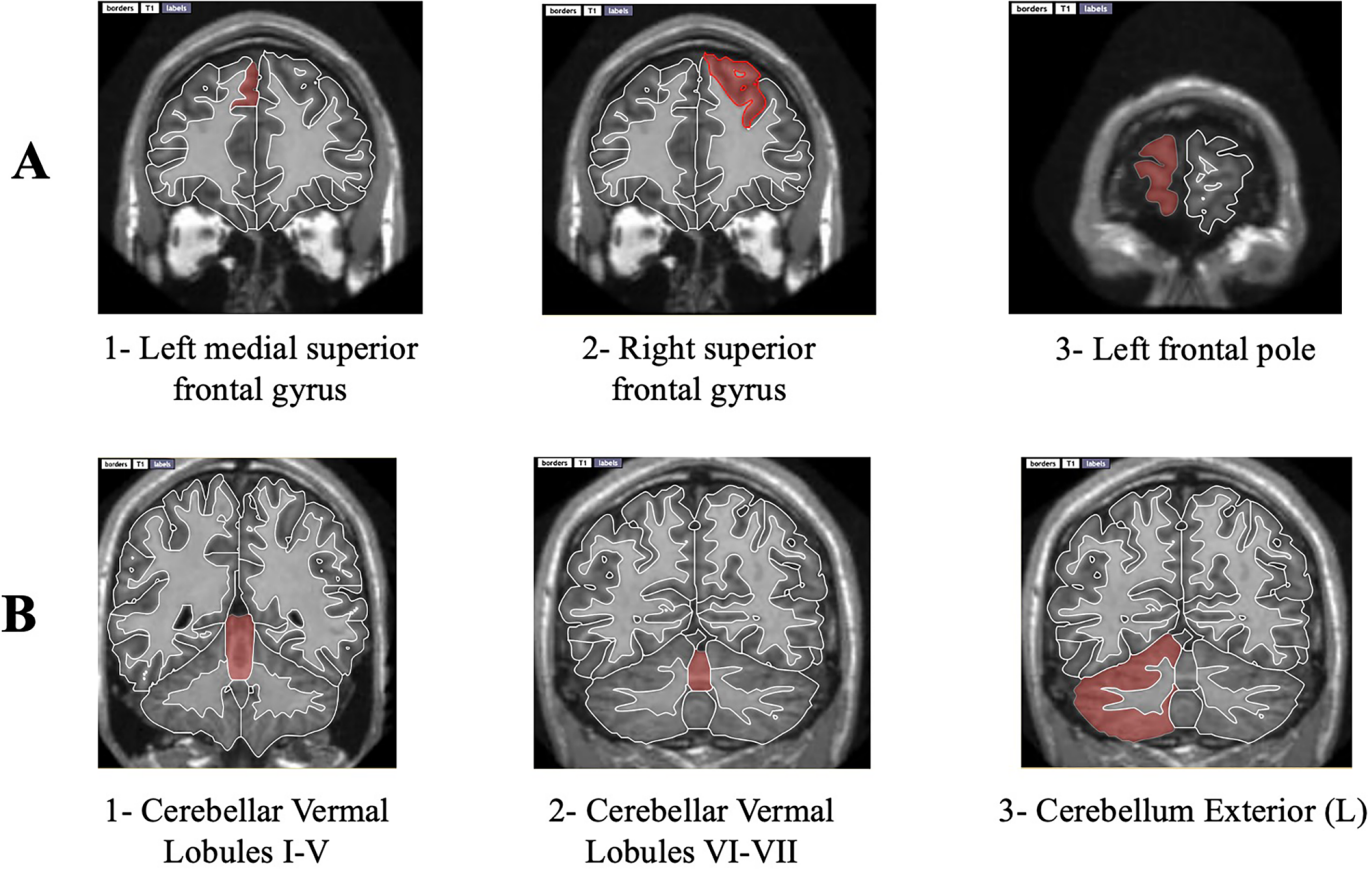
Top structural predictors of ketamine treatment response and non-response. Top neuroanatomical predictors of ketamine treatment response (**A**) and non-response (**B**), derived from a linear SVC model trained on regional gray matter volumes using the Neuromorphometrics brain atlas. Images were generated using atlas-based overlays. In (**A**), greater volume in the highlighted regions was associated with increased likelihood of treatment response. In (**B**), greater volume was associated with increased likelihood of non-response.

Increased GMV in frontal regions, including the left medial superior frontal gyrus, right superior frontal gyrus, and left frontal pole, was associated with a higher likelihood of response to ketamine. In contrast, greater volume in cerebellar regions, including cerebellar vermal lobules I–V, VI–VII, and the left cerebellar exterior, predicted non-response. These patterns are visualized in Fig. 2 and Fig. 3.

#### Mechanistic specificity assessment in saline-treated cohort

To evaluate whether the model signature was specific to ketamine, the trained classifier was applied to the independent saline-treated TRD cohort. Model performance declined, yielding a balanced accuracy of 41.1% and an AUC of 0.45. These values did not exceed chance performance (*p* = 0.88, permutation test). This lack of generalization supports the specificity of the model for ketamine-induced antidepressant effects, rather than nonspecific symptom improvement or measurement artifacts.

#### External validation in independent ketamine cohorts

The generalizability of the model was further tested in a pooled external validation cohort of ketamine-treated TRD patients drawn from geographically and methodologically distinct sites. Despite this heterogeneity, the model retained moderate performance predicting response in the external cohort (BAC 60.0%, sensitivity 66.7%, specificity 53.3%, AUC 0.65). Statistical significance was confirmed via permutation testing (*p* = 0.01). These results support the external validity of the model across geographically and methodologically distinct TRD cohorts. Validation performance is summarized in Table 2.

### Transcriptomic characterization of predictive brain regions

**Fig. 4:**
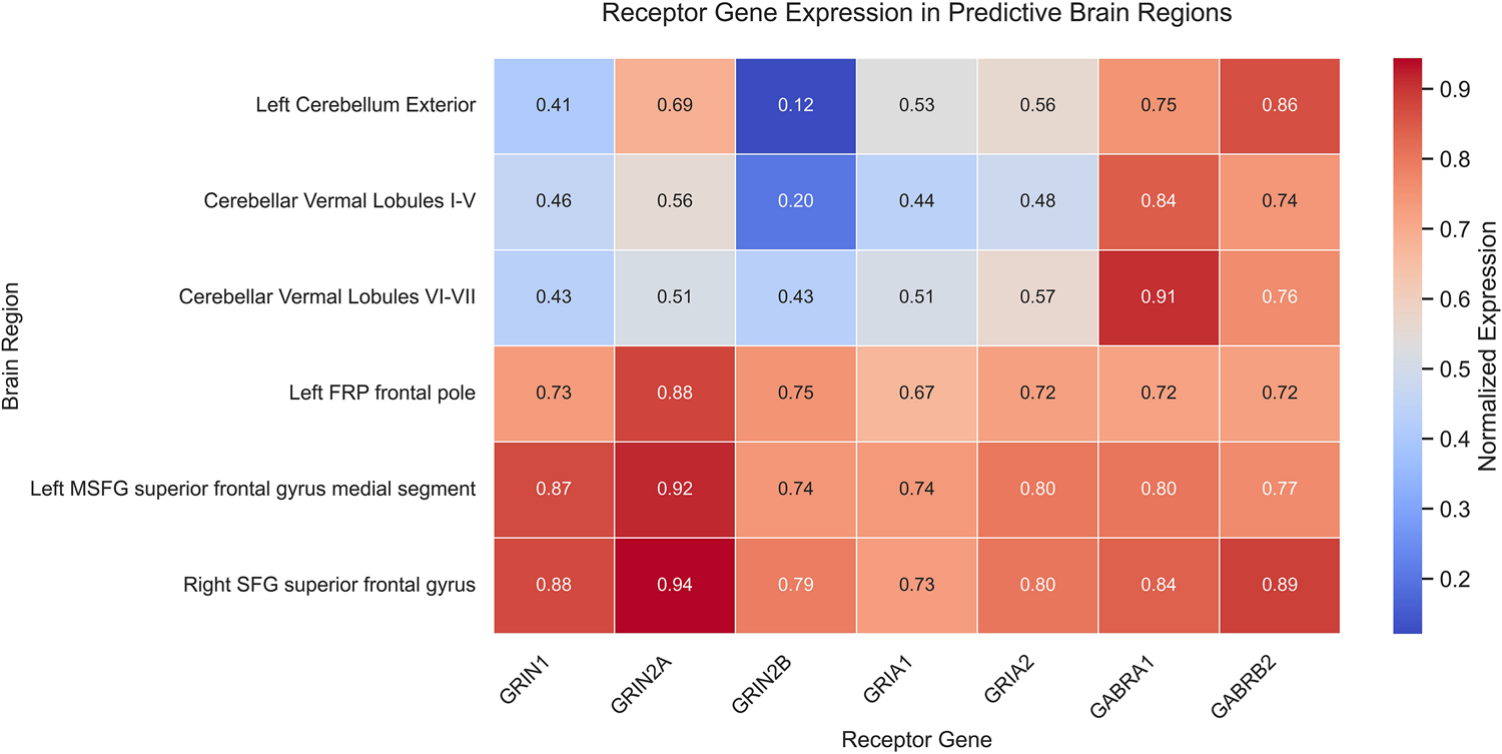
Normalized gene expression of ketamine-relevant receptors across top predictive brain regions, mapped using the Allen Human Brain Atlas (AHBA). This heatmap shows normalized gene expression values for seven receptor subunit proteins implicated in ketamine’s mechanism of action (columns) across six brain regions (rows) identified as the top predictors of treatment response from the ML model. Gene expression values were extracted from the AHBA using the abagen toolbox, which aggregates postmortem microarray data from six adult donors and maps it to the MNI space using the Neuromorphometrics atlas. The brain regions are ordered by ML-derived response relevance: The bottom three rows correspond to frontal regions (Left FRP frontal pole, Left MSFG superior frontal gyrus medial segment, Right SFG superior frontal gyrus), where greater gray matter volume predicted treatment response. The top three rows correspond to cerebellar regions (Left Cerebellum Exterior, Vermal Lobules I–V and VI–VII), where greater volume predicted non-response. Frontal regions showed consistently higher expression of NMDA receptor subunits (GRIN1, GRIN2A, GRIN2B), AMPA receptors (GRIA1, GRIA2), and GABA receptor subunits (GABRA1, GABRB2), relative to cerebellar predictors. Color intensity represents normalized AHBA expression values per gene-region pair, with numeric annotations indicating exact expression levels. This analysis was intended to provide biological contextualization of predictive regions using genes implicated in ketamine pharmacology, rather than to perform a genome-wide enrichment analysis of receptor gene expression.

Figure 4 displays normalized expression levels of ketamine-relevant receptor subunits across the six most predictive brain parcels identified by the ML model—three positively weighted (response-associated) frontal regions and three negatively weighted (non-response-associated) cerebellar regions. Frontal regions—where increased volume predicted treatment response—exhibited higher expression of NMDA subunits (GRIN1, GRIN2A, GRIN2B) and AMPA subunits (GRIA1, GRIA2) (range ≈ 0.7–0.9) relative to cerebellar regions, which predicted non-response and showed lower expression (range ≈ 0.2–0.5). Notably, GABA-_A_ subunit genes (GABRA1, GABRB2) were also elevated in frontal parcels, though differences were more modest.

## Discussion

### Prediction

#### Main findings

In this study, we developed an externally validated machine learning model that predicts short-term antidepressant response to IV ketamine in TRD patients using pre-treatment GMV data. We identified a multivariate neuroanatomical signature distinguishing ketamine responders from non-responders with a BAC of 72% in the original sample and 60% in an independent external cohort. This predictive performance substantially exceeds chance and is, to our knowledge, the first externally validated model for ketamine response in a TRD-only sample using structural imaging.

#### Relation to prior prediction work

Our work builds on prior studies that have sought biological predictors of ketamine’s antidepressant effects [24–26]. Consistent with the literature, our results support the idea that neurobiological heterogeneity underlies differential ketamine responses [27, 33]. Existing prediction efforts have largely relied on univariate associations or clinical markers, including symptom severity, inflammatory cytokines, or dissociative responses, which typically lack individual-level predictive power and fail to generalize across cohorts [24–26, 39, 51]. Even prior neuroimaging studies—often limited by small sample sizes or mixed MDD/TRD populations—report modest internal accuracy (~60–65%) and rarely attempt out-of-sample testing [40]. Moreover, most previous machine learning models do not focus on TRD specifically, despite this population carrying greater burden and clinical urgency [7, 52]. In clinical practice, ketamine remains administered largely by trial and error, with response rates showing high heterogeneity across patients and studies, including with repeated dosing, despite high costs, monitoring needs, and potential for dissociative or adverse effects [11, 19, 22, 53]. Our model demonstrates the potential to address this unmet need by using pre-treatment structural imaging to identify a reproducible neuroanatomical signature of response, offering a pathway toward more personalized, evidence-informed treatment selection in TRD.

#### Generalizability and mechanistic specificity of the model

External validation of our model demonstrated reasonable generalizability (60% BAC), despite inherent challenges such as heterogeneity in samples and MRI protocols, indicating the signature is not site-idiosyncratic. In contrast, the classifier failed to generalize to a saline/placebo cohort (BAC 41%), functioning as a negative control and supporting a ketamine-specific signal rather than a general prognostic marker. Together, these results increase confidence that the model captures pharmacologically relevant variance while illustrating the expected attenuation from discovery to external testing.

#### Clinical implications

Our results carry potential practical implications for the clinical management of TRD. At present, the findings show proof-of-concept evidence that a reproducible neuroanatomical signal exists across heterogeneous ketamine datasets. If replicated and translated into a user-friendly tool, an MRI-based ketamine response predictor could substantially improve treatment planning. In current practice, clinicians have little objective basis for choosing ketamine for one patient vs. another aside from clinical impression and prior treatment history. Our MRI-based predictive model could prospectively identify likely responders, helping clinicians prioritize ketamine for appropriate candidates and spare likely non-responders unnecessary infusion, monitoring, and side-effects. Many patients with TRD already undergo MRI during clinical work-ups or research participation; these routine scans could be repurposed to provide predictive information.

### Mechanistic insights

#### Main findings

Two neuroanatomical features emerged as particularly predictive of ketamine response: gray matter volume in the frontal cortex and cerebellum. Greater frontal cortical volume at baseline was associated with response, whereas increased cerebellar volume was characteristic of non-responders. These structural features offer mechanistic insight into the neurobiological substrates modulating ketamine’s antidepressant action.

#### Frontal cortex

Frontal cortical morphology has consistently been implicated in ketamine’s efficacy. The frontal regions identified as being important predictors of response overlap with dorsolateral and anterior prefrontal regions known to be important in the cognitive control of emotion; thought to be hypoactive in neurobiological models of depression; and used as targets in neuromodulation treatments such as transcranial magnetic stimulation, and direct current stimulation [54–57].

Mechanistically, ketamine induces a glutamate surge via NMDA receptor antagonism, which initiates synaptogenesis and restores connectivity in prefrontal circuits disrupted by chronic stress [58]. This cascade is thought to be driven in part by BDNF signaling and mTOR activation, facilitating rapid structural plasticity [13, 14, 49, 50, 59]. Patients with greater medial and superior frontal volume may have enhanced “neuroplastic reserve”—a denser or more intact neuronal architecture that better supports ketamine-induced remodeling. This interpretation aligns with prior findings associating higher pre-treatment activity or volume in frontal regions, including the rostral anterior cingulate, with superior clinical response to ketamine [31, 60].

#### Cerebellum

By contrast, the association of larger cerebellar volume with non-response presents a more novel and less intuitive observation. The cerebellum is increasingly being recognized for its role in mood, cognition, and emotion through bidirectional cerebello-cortical loops. Structural alterations in cerebellar lobules—particularly vermal and hemispheric subregions—have been reported in depression [61, 62]. In our study, TRD patients with non-response exhibited relative hypertrophy in these same regions. This finding may reflect a hypothesis-generating observation consistent with a maladaptive compensatory phenotype in which aberrant cerebellar morphology contributes to persistent network dysfunction.

#### Transcriptomic bridge

To explore potential molecular correlates of these structural findings, we leveraged transcriptomic data from the Allen Human Brain Atlas using the abagen toolbox [46–48]. Gene expression analysis revealed that frontal regions—those associated with response—exhibited higher expression of NMDA receptor subunits (GRIN1, GRIN2A, GRIN2B), AMPA subunits (GRIA1, GRIA2), and GABA-_A_ subunits (GABRA1, GABRB2) relative to negatively weighted cerebellar predictor regions identified by the model- patterns consistent with ketamine’s glutamate–AMPA–BDNF/mTOR cascade [49, 50, 63]. These results provide biological plausibility for our structural findings: frontal regions may serve as optimal substrates for ketamine’s pharmacodynamic effects, while enlarged cerebellar regions, with comparatively sparse receptor expression, may represent structurally dominant but functionally inert nodes within the antidepressant response network.

We therefore propose that cerebellar hypertrophy in non-responders may signify a morphologically entrenched and molecularly refractory circuit phenotype—potentially a high-gain, low-plasticity feedback loop that exerts sustained influence over medial prefrontal systems. In this model, ketamine’s cortical effects are counteracted by maladaptive cerebellar output, undermining antidepressant response. Meanwhile, patients with preserved frontal volume and higher receptor expression are structurally and molecularly positioned to benefit from ketamine-induced plasticity.

This dual-anatomical and transcriptomic framework extends current models of ketamine response and highlights the importance of considering cerebellar morphology not simply as a peripheral correlate, but as a potential determinant of treatment resistance in TRD.

### Strengths and limitations

This study’s strengths include, to our knowledge, being the first to demonstrate that structural MRI alone can support individual-level prediction of short-term ketamine response in a TRD-only cohort, with repeated nested cross-validation and independent external validation—addressing a gap noted in recent ketamine neuroimaging reviews and MRI prediction meta-analyses [27, 33, 64]. Methodologically, we combined whole-brain GMV parcellations, repeated nested cross-validation with permutation testing, external validation, and a saline negative-control generalization test—design choices aligned with best-practice recommendations for clinical prediction modelling [35, 65]. Finally, using pretreatment sMRI, a stable and broadly available modality with demonstrated multi-centre reproducibility, increases translational potential.

Limitations include modest sample sizes, particularly in the external and saline cohorts, widening uncertainty around point estimates. We tested 24-h outcomes only; whether baseline sMRI predicts durability of benefit remains unknown. Features were restricted to regional GMV; adding thickness, surface area, connectomic measures, and clinical/cognitive variables may improve performance. We did not apply inter-site harmonization to the external ketamine cohorts, as healthy control data were unavailable to support conventional group-based methods such as ComBat. While this may have contributed to variability, the model’s significant performance under these conditions supports its robustness and real-world transportability. Furthermore, participant-level psychiatric comorbidity information was not available across cohorts. We were, therefore, unable to evaluate whether comorbid conditions such as anxiety disorders influenced model predictions. Future studies with harmonized clinical phenotyping will be important to determine whether the observed neuroanatomical signature remains robust across diagnostic heterogeneity. Lastly, the post hoc imaging-transcriptomic analysis should also be interpreted cautiously, as the AHBA is based on six adult postmortem donors with incomplete regional sampling and does not reflect patient-specific gene expression.

## Conclusions and future directions

In this study, we present the first externally validated AI-based model of response to ketamine using machine learning models trained on pretreatment structural neuroimaging data. The model was specific to ketamine and implicated the superior frontal gyri, the left frontal pole, and the cerebellar vermal and lateral volumes in ketamine’s neurobiological mechanism of action. These findings provide initial evidence that readily obtainable baseline sMRI scans could inform individualized decisions about ketamine treatment, potentially shortening the protracted trial-and-error trajectory that typifies care in TRD. Our results show promise that an sMRI-guided decision-support tool could help clinicians prioritize likely responders, spare non-responders unnecessary exposure, and shorten time-to-benefit in TRD pathways. Future work should extend validation to additional, independent datasets, evaluate predictors of longer-term outcomes, and test whether incorporating complementary data types such as clinical, functional, or molecular measures further enhances prediction, and determine whether such models can inform treatment selection relative to other intervention.

## Supplementary information

Supplement

## Data Availability

Data were taken from previously conducted clinical trials (NCT03237286, NCT00088699, and NCT00768430). For details on the data and access requests, please contact the original study investigators, subject to appropriate data sharing agreements.
