# Supplementary material for: Structural imaging predictors of ketamine response in treatment-resistant depression: a machine learning approach
Source: Transl Psychiatry. 2026 May 12;16:330. doi: 10.1038/s41398-026-04085-4 (PMC13338048; doi:10.1038/s41398-026-04085-4)
Supplement: Supplementary file 1 — Supplement [file 41398_2026_4085_MOESM1_ESM.docx]

**Supplemental Content**

**Methods 1: Trial Descriptions**

**Discovery trial — NCT03237286: Intravenous Ketamine Plus Neurocognitive Training for Depression**

Randomized, double‑blind, placebo‑controlled single‑site trial in adults with treatment‑resistant depression (TRD). Participants received a single IV ketamine infusion (0.5 mg/kg over 40 min) or saline. Our discovery dataset included only ketamine‑arm participants with pretreatment T1 sMRI and MADRS measured at baseline and ~24 h post‑infusion (per main manuscript Methods); saline‑arm data were used for negative‑control analyses. Detailed protocol and dose/timing parameters are reported in the registry.

Psychiatric comorbidity considerations.

Participants met DSM-5 criteria for major depressive disorder and TRD. Individuals with bipolar disorder, psychotic disorders, autism spectrum disorder, or current problematic substance use were excluded. The trial protocol did not report comorbid anxiety or personality disorders within the imaging subset analysed here. Because harmonized participant-level comorbidity variables were not available across the included datasets, comorbidity status could not be incorporated into the prediction models.

**Registry link:** https://clinicaltrials.gov/study/NCT03237286.

**Primary publication**: Price RB, Spotts C, Panny B, et al. A novel, brief, fully automated intervention to extend the antidepressant effect of a single ketamine infusion. *Am J Psychiatry.* 2022;179(12):959-968. doi:10.1176/appi.ajp.20220216.

**External validation trial A — NCT00088699: Rapid Antidepressant Effects of Ketamine in Major Depression**

NIH/NIMH randomized, placebo‑controlled trial examining the rapid antidepressant effects of single‑infusion IV ketamine (typically 0.5 mg/kg over 40 min) in major depression. For external validation, we selected ketamine‑treated participants with pretreatment T1 sMRI and MADRS available at baseline and ~24 h. Where crossover designs were used, we aligned our extraction to the first ketamine session to avoid information leakage. Study overview and eligibility criteria are available on ClinicalTrials.gov; design and dosing details are also reported in the primary publication.

**Registry link:** https://www.clinicaltrials.gov/study/NCT00088699

**Primary publication**: Zarate CA Jr, Singh JB, Carlson PJ, *et al.* A randomized trial of an N‑methyl‑D‑aspartate antagonist in treatment‑resistant major depression. *Arch Gen Psychiatry.* 2006;63(8):856‑864.

**External validation trial B — NCT00768430**

Randomized clinical trial of single‑infusion IV ketamine (0.5 mg/kg over 40 min) in treatment‑resistant depression. For the present analysis, we included ketamine‑treated participants with pretreatment T1 sMRI and baseline/24‑h MADRS.

**Registry link**: https://clinicaltrials.gov/study/NCT00768430

**Primary publication**: Murrough JW, Iosifescu DV, Chang LC, et al. Antidepressant efficacy of ketamine in treatment-resistant major depression: a two-site randomized controlled trial. *Am J Psychiatry.* 2013;170(10):1134-1142. doi:10.1176/appi.ajp.2013.13030392.

**Methods 2: Sample Comparisons Across Cohorts**

To assess the comparability of the discovery and external validation cohorts, we conducted statistical comparisons of demographic and clinical variables. Participants in the external validation cohorts were significantly older than those in the discovery sample (Welch t-test: *t*(118.2) = −3.34, *p* = 0.001). However, there were no significant differences between the discovery and external samples in baseline depression severity as measured by MADRS (*t*(103.2) = 1.22, *p* = 0.226) or sex distribution (χ²(1) = 2.03, *p* = 0.15).

Within the external validation dataset, participants from the NY cohort were significantly older than those from the MD cohort (*t*(44.6) = −2.64, *p* = 0.011). However, the MD and NY cohorts did not significantly differ in baseline MADRS severity (*t*(45.7) = 1.05, *p* = 0.299) or sex distribution (χ²(1) = 1.67, *p* = 0.197).

Age and sex were also included as covariates in the machine-learning preprocessing pipeline, where their effects were regressed out from the imaging features prior to model training.

**MRI Acquisition Parameters for Discover Cohort**

Structural MRI data were acquired using two closely matched T1-weighted MPRAGE protocols on Siemens Prisma 3T scanners. Sequence parameters were highly similar across acquisition versions, with TR = 2400 ms, flip angle = 8°, inversion time (TI) = 1000 ms, and GRAPPA acceleration factor = 2. Echo time (TE) differed slightly between protocols (2.22 ms vs 3.02 ms).

The primary difference between acquisition versions was spatial resolution, with voxel size of approximately 0.8 × 0.8 × 0.8 mm versus 1.3 × 1.3 × 1.3 mm, reflecting differences in matrix and slice resolution.

These protocols therefore represent closely matched MPRAGE acquisitions with consistent contrast properties.

All images were processed using a standardized CAT12 preprocessing pipeline, including spatial normalization to MNI space, modulation, and smoothing, thereby ensuring that regional gray matter measures were derived in a common anatomical space and resolution framework across all scans. In addition, scanner/protocol-related variance was explicitly accounted for in the machine-learning pipeline by including a binary covariate, with its effects regressed out prior to model training. Together, these steps minimize the influence of acquisition-related differences on the resulting features and model performance.

**Methods 3: Negative‑control and reverse‑validation analyses**

**Primary negative‑control (reported in main text).**

To test pharmacologic specificity, the ketamine‑trained model was evaluated on the **saline/placebo** arm from the discovery trial; performance was at chance (BAC **41%**), consistent with the model capturing **ketamine‑specific** rather than general prognostic signal.

**Reverse analysis (saline‑trained → ketamine‑tested).**

As a complementary check, we attempted to train an analogous classifier in the saline arm and evaluate it externally in ketamine‑treated datasets (i.e., the reverse of our primary design). This analysis was not feasible due to the small size and severe class imbalance in the saline cohort (very few responders), which led to unstable or undefined stratified folds and outer folds with zero positive cases under our cross‑validation scheme. Because achieving stable estimates would have required aggressive resampling/oversampling that would not be comparable to our primary pipeline—and would risk optimistic bias—we did not proceed with a saline‑trained model. The negative‑control result reported above therefore serves as our specificity test, and we plan to revisit the reverse analysis once larger placebo datasets become available.

**Methos 4: Data & Software Availability**

The data used in this study are derived from previously published clinical trials and are not publicly deposited in a centralized repository. Data are available through the original study investigators or via ClinicalTrials.gov, subject to data sharing agreements and applicable institutional and ethical approvals. All machine-learning analyses were performed using NeuroMiner (v1.3; https://github.com/neurominer-git/NeuroMiner-1), an open-access platform designed for fully reproducible machine-learning workflows in neuroimaging. The Methods section provides a detailed description of the preprocessing pipeline, feature selection, model training, and validation procedures, sufficient to enable replication of the analyses.
